# Multi-photon, label-free photoacoustic and optical imaging of NADH in brain cells

**DOI:** 10.1038/s41377-025-01895-x

**Published:** 2025-08-07

**Authors:** Tatsuya Osaki, W. David Lee, Xiang Zhang, Rebecca E. Zubajlo, Mercedes Balcells-Camps, Elazer R. Edelman, Brian W. Anthony, Mriganka Sur, Peter T. C. So

**Affiliations:** 1 Picower Institute for Learning and Memory, Massachusetts Institute of Technology, Cambridge, MA USA; 2 Whitehead Institute for Biomedical Research, Massachusetts Institute of Technology, Cambridge, MA USA; 3 Department of Mechanical Engineering, Massachusetts Institute of Technology, Cambridge, MA USA; 4 Institute of Medical Engineering and Science, Massachusetts Institute of Technology, Cambridge, MA USA; 5 Department of Biological Engineering, Massachusetts Institute of Technology, Cambridge, MA USA

**Keywords:** Imaging and sensing, Photoacoustics

## Abstract

Label-free detection of biological events at single-cell resolution in the brain can non-invasively capture brain status for medical diagnosis and basic neuroscience research. NADH is an universal coenzyme that not only plays a central role in cellular metabolism but may also be used as a biomarker to capture metabolic processes in brain cells and structures. We have developed a new label-free, multiphoton photoacoustic microscope (LF-MP-PAM) with a near-infrared femtosecond laser to observe endogenous NAD(P)H in living cells. The imaging depth of NAD(P)H in tissues with all-optical methods is limited to ~100 μm in brain tissue by the strong absorption of the near-ultraviolet fluorescence. Here, acoustic detection of the thermal signature of multi-photon (three-photon) excitation of NAD(P)H, a low quantum yield fluorophore, allows detection at an unprecedented depth while the focused excitation ensures high spatial resolution. We validated the photoacoustic detection of NAD(P)H by monitoring an increase in intracellular NAD(P)H in HEK293T cells and HepG2 cells incubated in NADH solution. We also demonstrated the detection of endogenous NAD(P)H photoacoustic signals in brain slices to 700 μm depth and in cerebral organoids to 1100 μm depth. Finally, we developed and demonstrated simultaneous photoacoustic and optical imaging of NAD(P)H in brain cells with a real-time image acquisition and processing pipeline. This approach could open a new door to monitor brain metabolic changes during development and disease, and changes due to neuronal activity, at single-cell level deep in the brains of both humans and animals.

## Abstract (web summary)

Label-free, multiphoton photoacoustic microscope (LF-MP-PAM) with a near-infrared femtosecond laser to observe endogenous NAD(P)H of neurons in brain slices and cerebral organoids.

## Graphical abstract

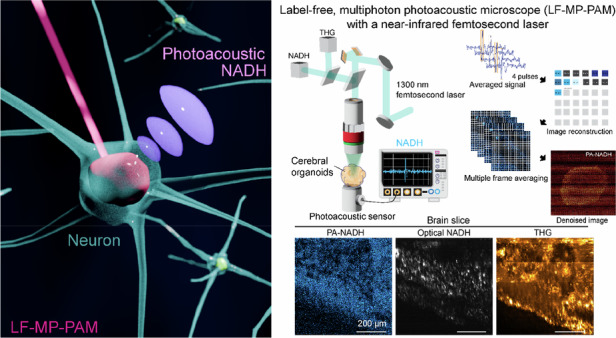

## Introduction

Photoacoustic (PA) imaging is an emerging modality that has generated increasing interest for its use in preclinical and clinical research^1,2^. PA imaging combines the benefits of optical excitation with acoustic detection, which transmits through brain tissue better than fluorescence emission, offering a deeper imaging modality compared to conventional imaging technologies^3^. Focused optical excitation in PA imaging provides single-cell level resolution and provides rich contrasts that reveal detailed anatomical, functional, and molecular information, such as detailed vascular structures^4^, hemoglobin oxygeneration^5^, and uptake of contrast agents^6^. Acoustic detection benefits from the minimal scattering of ultrasound waves in biological tissues and the brain, allowing PA imaging to acquire signals from deep within tissues. The ultrasound acoustic emission is created by the excitation of chromophores with an ultrashort laser pulse in biological tissues. A portion of photon energy is re-emitted as fluorescence. The non-radiative portion of the absorbed energy from the femtosecond pulses generates heat and thermal expansion within the focal volume since the thermal diffusion time constant is too long to dissipate the heat before thermal expansion. The thermal expansion initiates a propagating acoustic pulse at the focal zone, which can be detected at the tissue surface with minimal attenuation. The generated acoustic wave parameters are dictated by the excitation objective point-spread function, distribution of absorbing particles in the medium, and the thermal relaxation process. The photoacoustic waves are then detected by ultrasonic transducers and can be used to reconstruct the optical absorbance distribution in tissues^3^. This hybrid approach, in principle, would allow us to observe cells significantly deeper in the tissue than with all-optical imaging with label-free or fluorescent-based genetically encoded calcium indicators.

Nicotinamide adenine dinucleotide (NAD) is a critical molecule in the cellular metabolic pathway, which acts as a coenzyme in redox reactions and exists in two forms - oxidized and reduced (NAD(P)^+^ and NAD(P)H), respectively. The NAD(P)^+^/NAD(P)H ratio is maintained at certain levels depending on cell-type, tissue location, and their temporal status. This enzyme has been used as a label-free optical biomarker of metabolism due to its high and differential absorbance character (NAD^+^:250 nm, NAD(P)H:350 nm^7–9^). NAD(P)H dictates mitochondrial function, affects gene expression, relates to cell damage/death, and is correlated with calcium dynamics (neuronal spikes) in neurons in the brain^10–12^. During neuronal firing, NADH levels rapidly increase due to the citric acid cycle^7,13^, then convert back to NAD^+^ through the oxidation process. Therefore, optical absorption changes of NAD(P)H have been shown to reflect neuronal activity^14^. In addition, NAD(P)H has been shown to correlate with seizures^15^ and cortical spreading depression, which is involved in brain ischemia and migraine aura^16^, and is an early biological predictor for Alzheimer’s disease along with the aging process^10,11^. Therefore, real-time NAD(P)H detection in the brain could be a potential biomarker for estimating the dynamic activity of neurons during both normal function and disease progression^7,13,14,17,18^.

Endogenous NAD(P)H has utility as a potential label-free optical neuronal biomarker. It has been reported that the two-photon fluorescence imaging of NAD(P)H could provide the sensitivity and spatial resolution to resolve metabolic signatures in thin brain slices for studying the role of neuronal oxidative metabolism, followed by astrocytic glycolysis^18^. With its intimate relationship with cellular metabolism, it has found applications in numerous biomedical area ranging from personalized medicine in cancer to stem cell biology. However, it is not routinely used as an imaging target in neuroscience owing to the low quantum yield (of 5%) and its near-ultraviolet fluorescent emission (with emission maximum at about 450 nm) that is strongly absorbed and scattered in tissues. Today, all-optical imaging of NAD(P)H in brain tissue is limited to about 100-200 μm depth in brain tissue^17,18^. In addition, P-MRS^19^ (Phosphorus magnetic resonance spectroscopy) can detect NAD^+^ and NAD(P)H by exploiting the presence of a chemical shift between NAD and NAD(P)H. While P-MRS technology offers deep penetration, it has poor spatial resolution, typically on a millimeter scale. Therefore, no methods have been reported to measure endogenous and label-free NADH in deep tissue with single-cell resolution.

Photoacoustic detection of NAD(P)H could potentially offer deep imaging of NAD^+^/NAD(P)H. Owing to the low quantum yield of this enzyme, optical excitation induces a substantial thermal energy generation. Multi-photon excitation with a near-infrared (NIR) laser, along with photoacoustic detection, overcomes the limitations of observation depth and spatial resolution because the acoustic signal does not suffer from the orders of magnitude absorption and scattering of optical (fluorescent) emission signal through millimeters of brain tissue to the detector^20^. Photoacoustic detection of NADH with two-photon excitation has been previously reported, but in solution and gelatin tissue phantoms, not in biological samples^21^. To date, no study has reported endogenous NAD(P)H photoacoustic imaging with biological samples such as cells or brain slices in vitro.

In this study, we report label-free photoacoustic detection and imaging of endogenous NAD(P)H at the single-cell level in living cultured cells, brain tissue, and cerebral organoids utilizing our newly developed photoacoustic imaging system. The multi-photon photoacoustic configuration with a NIR-femtosecond laser (1300 nm) allows us to detect endogenous NAD(P)H in cells deep in tissue, such as brain slices and cerebral organoids, as the combination of multi-photon excitation and photoacoustic detection enhances the signal and overcomes the absorption/scattering^18^ limitation of conventional all-optical imaging. Cerebral organoids are a 3-dimensional human tissue in vitro model typically from induced pluripotent stem cells (iPS cells) or embryonic stem cells (ES cells)^22,23^. Cerebral organoids have been shown to recapitulate human brain developmental processes, including normal and pathological processes, using patient-derived iPS cells in vitro, and they show complex neuronal network activity derived from synaptic connections and plasticity^24–26^. Therefore, live observation of biological events in organoids importantly complements observations in vivo mice brain and human brain. In our approach, we first confirmed the NADH photoacoustic signal with standard NADH imaging of multiple concentrations in gels to validate the character of photoacoustic energy, frequency, and acoustic transit time. We then exogenously introduced NADH in cells by incubating HEK293T cells and HepG2 cells with NADH and observed an increase in photoacoustic signals, which we independently confirmed by fluorescent-based conventional NAD(P)H sensors. We further demonstrated that PA detection can overcome the current limitations of all-optical-based NAD(P)H detection by performing photoacoustic detection of NAD(P)H in thick tissue (700 μm in mouse brain slices and 1100 μm in cerebral organoids from human iPS cells). Lastly, we developed an imaging subsystem and integrated it into the photoacoustic and multiphoton laser platform to create photoacoustic images to demonstrate a photoacoustic-generated spatial map of NAD(P)H in organoid and brain slice cells. Our results and approach demonstrate the capability to image brain metabolic changes and neuronal activity at greater depths than currently possible with all-optical imaging for endogenous NAD(P)H.

## Results

### Multiphoton, label-free photoacoustic imaging system with a femtosecond laser

**Fig. 1:**
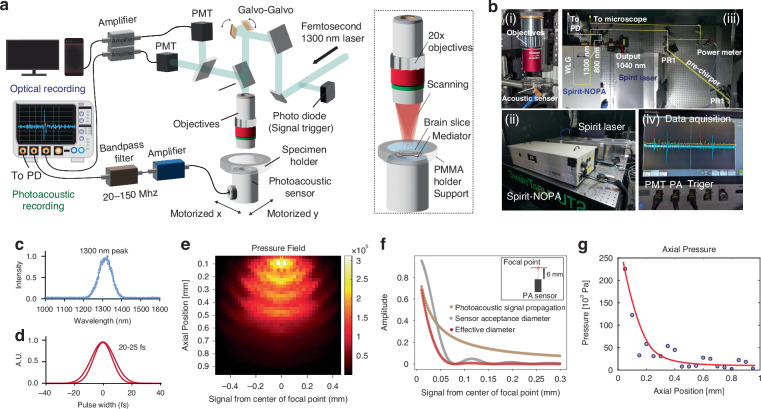
Setup of multiphoton. label-free photoacoustic detection system with a near-inferred (NIR) femtosecond laser. **a** The PMT signals and acoustic signals are displayed and recorded on the oscilloscope saved for post-processing. **b** A view of the objective above the specimen and the acoustic sensor below the specimen. The specimen is held in a 6 mm diameter and 6 mm deep saline well. A photo of the oscilloscope during the test in NADH solution showing the photoacoustic spikes in blue. Also shown is the optical PMT signal in yellow. The laser pulse repetition rate was set to 100 kHz. **c**, **d** Measured wavelength and pulse width of laser from the spectrometer after beam conditioning. **e** K-Wave simulation result of the acoustic pulse pressure field based upon the focal volume of 3P pulse created by the 0.4 NA objective at 1 mm depth and 25 mw laser power. **f** A simulation of the photoacoustic signal, the acoustic sensor near field acceptance angle at 6 mm from the sensor, and the combination of the two for the effective field of view (2x lateral distance) yielding an acceptance diameter of about 100 μm, which agrees well with the experimental observations. **g** The simulation of the expected pressure field as a function of the axial position

To deliver photoacoustic energy in deep tissue with a single-cell spatial resolution, we developed a new photoacoustic microscope with a femtosecond laser that utilizes multi-photon excitation (Fig. 1a–d, supplementary fig. 1a). For simultaneous detection of optical and photoacoustic signals an acoustic transducer was integrated into a multiphoton microscope below the objective and underneath a specimen (Fig. 1a). We exploited three-photon excitation of NAD(P)H at 1300 nm, instead of two-photon excitation at 800 nm, in order to take advantage of the significantly reduced tissue attenuation at the longer wavelength^21^. Ultrashort laser pulses (300 fs, 400 kHz, 16 W Fig. 1c) at 1300 nm were produced by a noncollinear optical parametric amplifier (NOPA) driven by a 1040 nm pump laser. Pulse widths were further tuned by a two-prism based pre-chirp system down to 20–30 fs by a two-prism pre-chirp system (Figs. 1d, S1a). The conditioned laser beam was scanned by a pair of galvo-galvo scanners to image and deliver energy for photoacoustic generation on the back aperture of the objective using a pair of custom-designed scan and tube lenses (Fig. S1b). According to the NAD(P)H absorbance one-photon spectrum, we estimate 1100–1400 nm as optimal for three-photon absorption. We further validated this wavelength by sweeping the wavelength through a NAD(P)H sample solution.

The Spirit-NOPA system (Fig. 1b) was chosen as it offered a variable repetition rate from 1 Hz to 400,000 Hz and a high pulse energy (170 nJ) needed for deep penetration. Based on acoustic and optical simulations of three-photon point spread function and heat relaxation process, we selected an objective with NA = 0.4, which would produce a 2.2 μm lateral (Fig. S1c) and 30 μm axial heated focal volume, an elongated prolate spheroid. Since the center acoustic frequency generated is inversely proportional to the radius of the heated volume, this focal volume size was selected to be small enough to yield cellular resolution (typical neurons are 5-15 μm in dimension in the brain) but not too small as to generate excessively high frequency acoustics pulses that would suffer strong tissue attenuation. Using K-Wave functions in MATLAB (Fig. S1d) an acoustic peak frequency of 90–125 MHz in the axial direction was predicted for our heated volume. While this acoustic frequency is much higher than typically used for ultrasound imaging in the brain, the expected attenuation through 2.7 mm (the average thickness of the human cortex) is −4 dB^27^ or a 44% loss which is insignificant compared to the 1/r-pressure loss (2 × 10^−3^) inherent from the 2 μm focal volume out to the transducer potentially 2.7 mm away. As a comparison, the NAD(P)H fluorescence signal at 450 nm has an extinction length of 50 μm and will attenuate by 3.5 × 10^−24^ through an equivalent 2.7 mm-thick tissue. We used commercially available acoustic transducers (125 MHz, element size: 0.125 inches in diameter), and 75 MHz acoustic transducers for selected experiments (Fig. S1d). The K-wave propagation model for the rectangular focal (heated) volume yields the predictions (Fig. 1e–g). The transducer was exactly aligned to the center of the objective to maximize the photoacoustic signal since the detection diameter in the near field is approximately 100 μm (Fig. 1g).

### Photoacoustic detection of NAD(P)H in gel tissue phantom

**Fig. 2:**
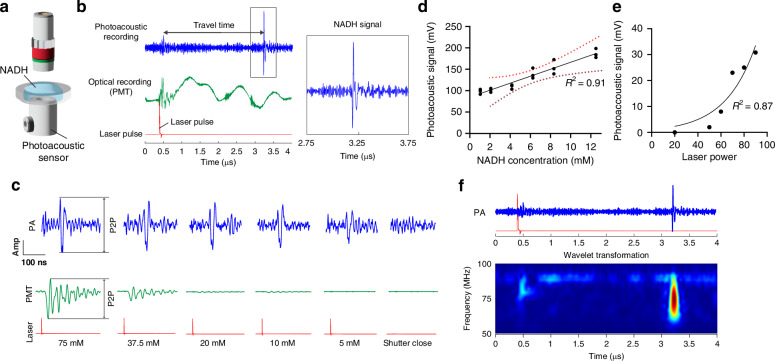
Characterization of 3-photon photoacoustic acoustic generation in NADH gels at varying concentrations. **a** Configuration of the NADH in the gel tissue phantom experiment. **b** The photoacoustic signal (blue) after a laser pulse (red) occurs 2.6 us later, which the acoustic delay built into the ultrasound transducer. **c** The acoustic signal (blue), optical signal (green) as the NADH solution at 5, 10, 20, 37.5, 75, and 150 mM. **d** The optical signals vs NADH concentration at 1, 2, 4.6, 9.3, and 18.75 mM. The linear concentration confirms that we are measuring NADH. The photoacoustic signal was present at 1/20 the concentration floor of the optical system, demonstrating the high sensitivity. The photoacoustic signal vs concentration shows a good linear fit with R = 0.91. **e** Photoacoustic signal vs laser power showing the cubic nature of the signal, thus confirming 3-photon excitation. Non-linear model (Exponential growth). R2 = 0.88. **f** The frequency analysis (wavelet transformation) of the pulse showing frequency around 88 MHz as predicted by the focal volume

In the NAD(P)H gel tissue phantom, we confirmed that the 1300 nm laser is absorbed by NAD(P)H, producing 450 nm fluorescence emission and generating a photoacoustic signal. NADH gel specimens were created and placed on top of an acoustic sensor (Fig. 2a). Laser pulses at 1300 nm was delivered by a 0.4 NA objective while the fluorescence signal was collected in an epi-geometry by a photomultiplier tube (PMT) after a 440-510 nm band-pass filter. Photoacoustic signals were amplified by the pulser receiver system and recorded by an oscilloscope. Photoacoustic signals from NADH were captured with the expected delay time of the built-in silicon delay element in the transducer. According to the specification of the acoustic transducer, we expected a 2.6 μs delay, and we indeed observed this delay from the laser trigger (Fig. 2b).

We first tested concentrations of 5, 10, 20, 37.5, 75, and 150 mM of NADH solution and observed a concentration-dependent signal strength change of photoacoustic and PMT signal (Fig. S2a, b, Fig. 2c). We observed a non-linear relationship of photoacoustic signals in higher concentrations (Supplemental Fig. 2a). We further tested NADH in gel from 0–12 mM of concentration, which should be biological range of NAD(P)H in neurons^18^ (Fig. 2d). A linear relationship between NADH concentration and acoustic signal was observed (Fig. 2d). In addition, we varied the laser power and measured the PA signal (Fig. 2e). The cubic power dependence (Fig. 2e) of the photoacoustic signal confirmed three-photon excitation though we were limited to a narrow power range by the noise floor (6 mW) and the maximum laser power (25 mW). Time-frequency analysis based on wavelet transformation revealed that the NADH photoacoustic signal exhibited a frequency of approximately 60-90 MHz (Fig. 2f) which is consistent with the relationship between focal volume and acoustic frequency^28^, but it is slightly lower than an acoustic peak frequency of 90–125 MHz predicted by the k-wave simulation (Fig. S1d) which could be due to underestimating the heat volume and the non-ideal sharp edges of the simulated heated volume both of which would raise the predicted frequency. These results implied that our photoacoustic system, combined with NIR laser and acoustic transducer could successfully capture photoacoustic signals in a NADH standard sample.

### The photoacoustic signal increases with exogenous NADH uptake in cultured cells

**Fig. 3:**
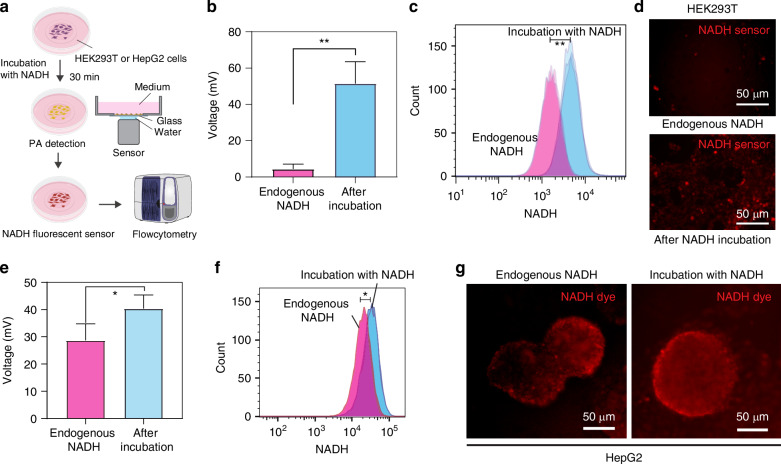
Detection of photoacoustic signal of exogenous NADH uptake. **a** Experimental setup of NADH incubation with HEK293T cells and HepG2 and photoacoustic detection, followed by flow cytometry with NAD(P)H fluorescent sensor. Before PA detection, cells were incubated with NADH in a culture medium for 30 min. **b** Photoacoustic signal of NADH before and after incubation, indicating the increase of NADH concentration in HEK293T cells. **c**, **d** Flow cytometry and microscopy observation with NAD(P)H sensor also showed the increase of NADH by uptake of NADH. **e** Photoacoustic signal of NAD(P)H before and after incubation, indicating the increase of NAD(P)H concentration in HepG2. **f**, **g** Flow cytometry and microscope observation with NAD(P)H sensor showed the increase of NAD(P)H by uptake of NADH in HepG2. *p < 0.05, **p < 0.01, Student’s t-test

We next demonstrated the detection of photoacoustic signal of exogenous NAD(P)H in living cells with independent confirmation with a NAD(P)H fluorescent sensor^29–32^ (Fig. 3a). HEK293T (human embryonic kidney cells 293T) and HepG2 cells (hepatocellular carcinoma cells) were incubated with 100 μM of NAD(P)H solution for 30 min, which allowed the cell to uptake NAD(P)H, and then photoacoustic measurement was performed. NADH is taken up by cells through P2X7 receptors^33,34^ and hemichannels of connexin-43^35^. In HEK293T cells, the photoacoustic signal of NADH increased significantly, by 5-fold, during the incubation (Fig. 3b). Subsequent analysis with a NAD(P)H fluorescent sensor by flow cytometry (Fig. 3c) and fluorescent microscopy (Fig. 3d) also indicated an increase of NAD(P)H concentration levels, consistent with uptake of exogenous NAD(P)H. Like HEK293T cells, we also observed increased NADH after incubation with NAD(P)H in HepG2 cells using both photoacoustic measurement (Fig. 3e) and flow cytometry with NAD(P)H sensor (Fig. 3f, g). In conjunction with the NADH measurements in gel tissue phantom and NADH fluorescent sensor assessment, we conclude that the photoacoustic signal in these cultured cells is predominantly derived from endogenous and exogenous NAD(P)H.

There are several other potential chromophores, such as FAD, NAD(P)H, collagen, and tryptophan (Fig. S3), which absorb light at 1-photon wavelengths near the 1-photon NA(P)H absorption and can be assumed to have a 3-photon absorption near 1300 nm. However, the concentration of FAD and NAD(P)H is an order of magnitude less than NADH and would not be confounding factor^13,36,37^. Based upon the foregoing incubation test, standard concentration test and the comparison with other molecules would strongly suggest that the signals derive from NAD(P)H.

### Endogenous NAD(P)H optical and photoacoustic measurement in mouse brain slices

**Fig. 4:**
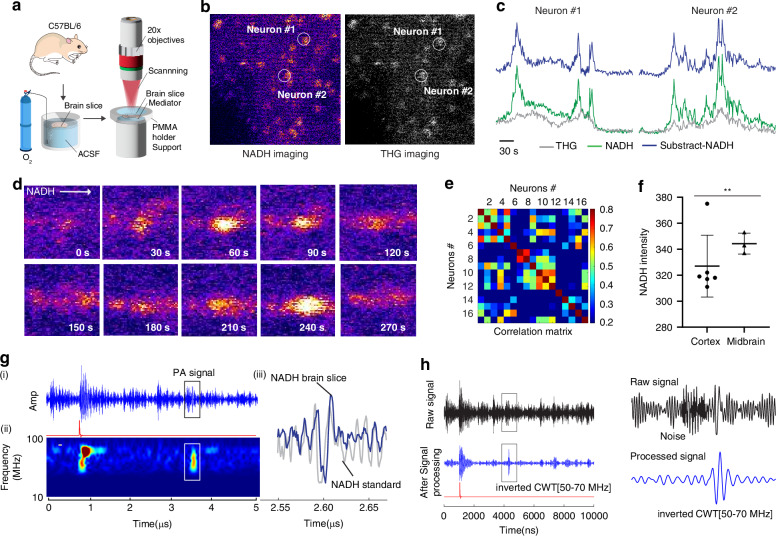
Demonstration of single neuron NAD(P)H optical imaging with a 3-photon microscope. **a** Wild type mouse brain slice placed in the 3 photon microscope. **b** NAD(P)H in the all-optical NADH 475–575 nm (525 nm center) channel (left) and the THG 390–410 nm (400 nm center) channel (right). **c** NAD(P)H and THG signals vs time individually and the difference of the two showing good concordance of the signal at a neuron as indicated by the THG signal. **d** Representative time-lapse images of optical NAD(P)H. **e** Correlation between neurons from the NAD(P)H signal. **f** NAD(P)H signal intensity as a function region of the brain. *n* = 3 different brain slices. **g** Top: Raw acoustic signal indicated as the expected time delay of 2.6 µs set by the silica delay line built into the transducer. Bottom: continuous wavelet transformation of the signal showing the expected frequency between 50–90 MHz. **h** A comparison of the photoacoustic raw signal (top) and after continuous wavelet transform (cwt, bottom) to extract the 50–70 MHz signal. **p < 0.01, Student’s *t*-test

Next, we performed optical and photoacoustic measurements of NADH in cells in mouse brain slices (Fig. 4a). Single cells could be observed with both the NADH signal from the photomultipiler (PMT) with a 440–510 nm band-pass filter and the third harmonic generation (THG) signal with 390–410 nm band-pass filter (Fig. 4b). THG signal generation is proportional to the cube of the refractive index mismatch between different materials. Therefore, interfaces between materials with substantially different refractive indices yield a strong THG signal. This sensitivity to interfaces allows THG imaging to provide high contrast for structures like cell membranes, lipid droplets, and other interfaces within cells and tissues, allowing us to obtain the morphology of cells. We first confirmed that the objective (NA: 0.4) had sufficient resolution to identify single neurons in GCaMP6s-expressing brain slices (Fig. S4b) with the same setup as used for previous measurements (Fig. S1a). The contrast and resolution were slightly lower compared to high NA objectives (NA: 1.05 objectives, Fig. S4b), but we could readily detect neuronal activity by tracking the changing calcium concentration in neurons due to electrical activity (Fig. S4c, d), which also demonstrated expected correlations between nearby neurons (Fig. S4e).

The time-lapse optical recording of NADH signals with objective (NA: 0.4) showed active cells in brain slices as they generated changes in NADH optical intensity (Fig. 4c, d). Our results correspond well with the published dynamic behavior of NADH in neurons and astrocytes (we did not distinguish neurons from astrocytes in this work), in which the NADH fluorescent response to electrical stimulus showed a strong temporal correspondence to intracellular Ca^2+^ increases^17,18^. The NADH intensity increased over a 60-second period and then declined in a cyclic manner (Fig. 4d). The time correlation matrix of the brain slice neurons showed a high temporal correlation among the neurons (and astrocytes) based on NADH signals (Fig. 4e). We also confirmed the variation in the NADH concentration in different brains regions, with higher levels of NADH in the midbrain region than the cortex (Fig. 4f), which is consistent with previous findings^38^.

The acoustic signal from brain slices of wild type mice (Fig. 4g(i)) appeared approximately 2.6 μs after the laser pulse, as was the case in the NADH gel phantom tissue experiment (Fig. 2). The wavelet transformation of the signal (Fig. 4g(ii)) indicated an acoustic signal at about 60-90 MHz as expected. The pulse shape represented a similar waveform as the NADH standard sample (Fig. 4g(iii)). Averaging the acoustic signal and inverted continuous wavelet transformation for a 60-90 MHz signal allowed us to increase signal-to-noise ratio, resulting in a pulse after the trigger which corresponded to the acoustic wave travel time plus the transducer delay (Fig. 4h). The photoacoustic signal/noise ratio of 9.7:1 (194 mv/20 mv) was recorded on an oscilloscope, demonstrating the sensitivity of the approach to investigate the NAD(P)H status of cells in the brain. Furthermore, we tested the change of NADH baseline level of brain slice with reduced oxygen for 24 h (Fig. S5), by optical NAD(P)H (Fig. S5a), photoacoustic NAD(P)H (Fig. S5b), and NADH sensor measurement (Fig. S5c). We confirmed that the three separate methods consistently showed an increase in NAD(P)H level, as reported in previous literature^39^. Together, these results suggest that we have successfully demonstrated endogenous NADH detection in neurons and astrocytes in brain slices with a photoacoustic signal from our system.

### Deep photoacoustic recording to thick brain slice and cerebral organoids

**Fig. 5:**
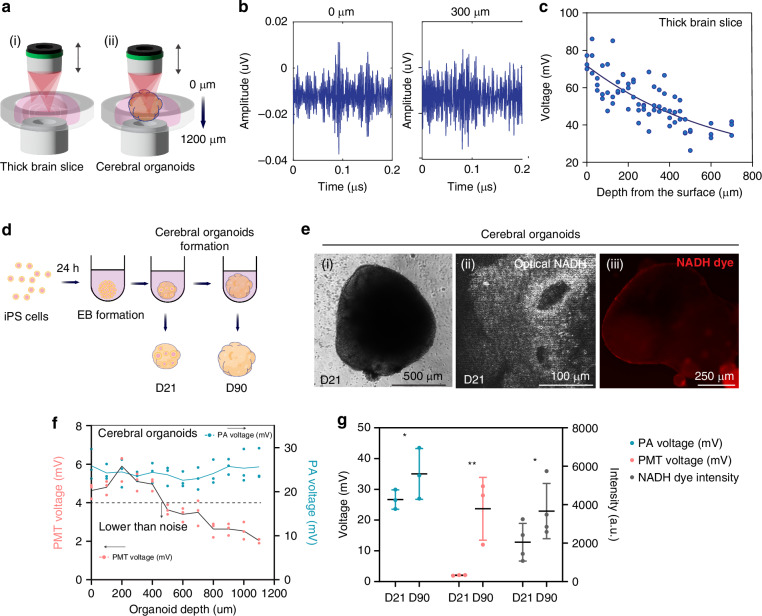
Deep tissue photoacoustic recording of NAD(P)H in brain slice and cerebral organoids. **a** Set up of brain slice and cerebral organoids (1.2 mm diameter). **b** Raw acoustic traces at 0 and 300 µm of depth, showing little signal loss. **c** The peak of the average acoustic signal from 0 to 700 μm of depth at a constant excitation power of 53 mW at the objective. **d** Human iPS cells were seeded to round-bottom 96 well plate to make embryonic bodies, and then, the cells were cultured for 21 days to obtain immature organoids and for 90 days to obtain mature organoids. **e** Cultured cerebral organoids from human iPS cells on day 21 as (ii) bright field image (ii) THG imaging of organoid structure at 1300 nm, and (iii) NAD(P)H sensor with a fluorescent microscope. **f** Cerebral organoid showing no loss in photoacoustic signal out to 1100 µm depth which was the total thickness of the organoid. The optical signal declined down to the noise floor at 400 µm depth. **g** Photoacoustic NAD(P)H (in mV), optical NAD(P)H (in mV), and fluorescent NAD(P)H (in intensity) in sensor showed a consistent increase in organoids at day 90 compared to day 21. *p < 0.05. **p < 0.01, Student’s *t*-test

The key conceptual and expected advantage of photoacoustic imaging is that it can exceed the current limit of deep tissue optical imaging. To demonstrate the capabilities of our multi-photon photoacoustic microscope system, we conducted three-photon photoacoustic measurement of NAD(P)H to 700 μm-depth from the brain slice surface in a thick brain slice (Fig. 5a(i)) and to 1100 μm depth in cerebral organoids (Fig. 5a(ii)). One-millimeter-thick brain slices from wild type mice were prepared to recapitulate thick tissue and enabled us to sweep the scanning from 0 to 700 μm of depth (Fig. 5c). In this thick brain slice, robust photoacoustic signals of NADH at multiple depths were observed with a high signal-to-noise ratio. We observed depth-dependent decay of photoacoustic NAD(P)H (Fig. 5c), showing the expected nonlinear relationship of the curve due to absorption and scattering at constant laser power input with a 1/e extinction length of 250 μm. Furthermore, we tested photoacoustic depth capability in cerebral organoids. We utilized human iPS-derived cerebral organoids at two different culture periods (day 21 and day 90) post-differentiation. Formation of iPS spheroids and differentiation protocols were as previously described^40^ (Fig. 5d, e). We measured both optical NAD(P)H and photoacoustic NAD(P)H signals as well as NADH fluorescent sensor signals in the organoids (Fig. 5e(ii) and (iii)). We observed a decline of optical NADH signal to 400 μm of tissue depth, which was the noise floor of the optical detection. We observed no signal decline of photoacoustic NADH through the entire depth (1100 µm) of the organoid sample (Fig. 5f). 1100 µm exceeds the imaging depth limit of 700 µm reported in other engineered tissue^41^ which like organoids have less scattering and absorption than brain tissue. In addition, we observed an organoid age-dependent increase in NADH from day 21 to day 90 using each of the three separate methods (Fig. 5g).

These optical and photoacoustic experiments demonstrate that we successfully observed endogenous NAD(P)H in thick brain slice and cerebral organoid. Furthermore, they confirm that photoacoustic NAD(P)H measurement could push the observation limitation of deep tissue beyond all-optical NAD(P)H measurement.

### Development of a photoacoustic imaging system for NAD(P)H

**Fig. 6:**
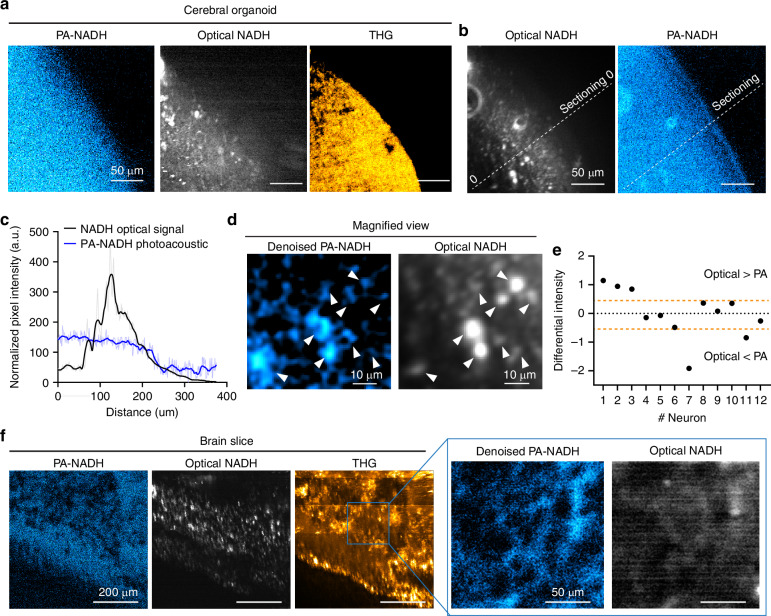
NADH photoacoustic image of cerebral organoids and brain slice. **a** NAD(P)H photoacoustic imaging of 3-month-cultured cerebral organoids (PA-NAD(P)H) along with THG and optical NADH. Wavelength: 1300 nm, laser rep rate: 200 kHz, acquisition frequency 2.0 GHz, dwell time: 20 μsec, 256 × 256 pixels, 0.76 frame/sec, laser power: 10–20 mW. Pixel resolution is 0.9 μm/pixel. **b**, **c** PA-NAD(P)H signals have little decay in the core of the cerebral organoid over the optical NAD(P)H signal. **d**, **e** Denoised image of label-free PA-NAD(P)H and magnified view showing PA-NAD(P)H signal at single cell resolution, overlapping optical NAD(P)H signal. **f** Photoacoustic image of NADH with optical-NADH and THG image in a brain slice. Pixel resolution is 2.1 μm/pixel

Lastly, we advanced the photoacoustic imaging system (LF-MP-PAM) beyond the oscilloscope-based system, enabling spatial imaging instead of just signal-averaged values of photoacoustic NAD(P)H, as demonstrated earlier (Figs. 2–5). To achieve real-time image creation, we implemented real-time signal conditioning, synchronized laser pulses with galvo-scanner movements and pixel recording, and modified the ScanImage software (see the Methods section and Fig. S6a). These modifications allowed us to define a narrow time window after the expected time delay from a laser pulse and calculate the average analog values for pixel intensity in the image (Fig. S6b). First, NAD(P)H standard samples were prepared with hair to provide spatial features, alongside optical THG imaging at the same location (Supplemental Fig. 6b, c). We then set a narrow acquisition time window (Fig. S6d) based on the laser trigger, resulting in the generation of images (Fig. S6d, e). The photoacoustic NAD(P)H images were consistent with the THG images (Fig. S6e). Subsequently, we imaged a cerebral organoid using THG, optical NAD(P)H, and photoacoustic NAD(P)H signals (Fig. 6a) at a depth of 100 μm, demonstrating a consistent overlap of signals (Fig. S6f). While optical signal strength decreased toward the center as organoid thickness increased, the photoacoustic signal remained robust with depth (Fig. 6c). These imaging results and their quantification reconfirmed that the photoacoustic NAD(P)H signal in the organoid was stronger than the optical NAD(P)H signal (Fig. 6b, c), as demonstrated in the previous section (Fig. 5e). Additionally, post-denoising processing and image deconvolution identified cellular images in the cerebral organoid from the photoacoustic NAD(P)H images (Fig. 6d, e), consistent with the optical NAD(P)H images. We also tested the imaging capability on a brain slice (Fig. 6f). The quantified differences in signal intensity between optical and photoacoustic NAD(P)H in single cells showed a strong correlation.

Therefore, our label-free photoacoustic and optical imaging system using an NIR laser is capable of monitoring and imaging endogenous NAD(P)H in cells and neurons located in deeper layers, achieving single-cell resolution. This LF-MP-PAM approach can be applied not only to measure NAD(P)H in in vitro tissues, such as brain slices and cerebral organoids, but also to detect other biomarkers in vivo.

## Discussion

We developed a novel label-free, multiphoton photoacoustic detection and imaging system (LF-MP-PAM) leveraging femtosecond laser excitation, which represents a significant advancement in imaging techniques for biological tissues. Our study successfully integrated three-photon excitation for NAD(P)H detection within a multiphoton photoacoustic microscopy framework, enabling high-resolution imaging of deep tissue structures. This approach addresses the longstanding challenges of traditional all-optical imaging techniques, which are often hindered by light scattering and absorption in complex biological samples. We first validated the photoacoustic system in cultured cells (HEK293T and HepG2) and illustrated the feasibility of detecting exogenous NADH uptake, corroborated by conventional NAD(P)H fluorescent sensor with flow cytometry and optical NAD(P)H imaging (Fig. 3). The significant increase in photoacoustic signals following NADH incubation supports the notion that our method can effectively monitor baseline level of NAD(P)H in live cells with a photoacoustic signal. In this observation with cells, other potential candidates with similar absorbance characteristics were considered (Fig. S3) as they might obscure the NAD(P)H signal. According to 1-photon or 2-photon excitation of molecules, FAD, NAD(P)H, and collagen are potentially chromophores at around 1300 nm. FAD-like NAD(P)H is involved in cellular metabolic processes. Collagen is an extracellular matrix, functions as a scaffold for cells and is secreted by cells to form a cellular microenvironment. However, the concentrations of FAD ^13^in cells are an order of magnitude less than NAD(P)H in cells and would not be a confounding factor^9^. In addition, other optically active molecules in cells, such as (3-photon) pyruvate and (2-photon) cytochrome C oxidase, absorb at different wavelengths than NAD(P)H (700–800 nm) and would not interfere. According to the 1-photon absorption of NAD^+^ at approximately 250 nm, we can estimate that they would be absorbed by 750 nm (3-photon), 1000 nm (4-photon), and 1250 nm (5-photon), but 4 and 5-photon excitation can be ignored due to insufficient laser power. Together with this literature, the effective concentration of other candidate molecules in cells, and our measurements, we conclude that NAD(P)H is a primary photoacoustic signal source in brain cells and cerebral organoids at 1300 nm with our system (Figs. 5 and 6).

Furthermore, we demonstrated the capability to measure endogenous NAD(P)H levels in mouse brain slice with both optical (Fig. 4b–e) and photoacoustic measurement (Fig. 4f–h), also revealing brain-region specific baseline NAD(P)H level by photoacoustic measurement (Fig. 4f). Most importantly we achieved a strong photoacoustic NAD(P)H signal at 700 μm depth in brain slices (Fig. 5b, c) which is 6-times greater than the optical imaging depth of NAD(P)H reported in the literature^13,42^. In cerebral organoids, we recorded a strong photoacoustic signal to at least 1100 μm depth (Fig. 5d–f), In addition, we observed an increased NAD(P)H baseline level in 3-month cultured cerebral organoids vs 3-week-old. During this period, cells typically differentiate from mitotic to post-mitotic cells along with the differentiation and maturation of neurons, and this transition is reflected in the change of NAD(P)H in growing cerebral organoids.

Simultaneous photoacoustic and all-optical imaging further reinforces the claim of NADH imaging at single cell resolution (Fig. 6c–e). Signal acquisition speed in the photoacoustic system compared to the optical system is defined by the signal traveling time of the optical signal and the acoustic signal to the detectors, and the scanning dwell time. The travel time of light and photoacoustic waves is around 3.3 ns (sample to PMT) and 330 ns (sample to acoustic sensor) for the optical and PA systems, respectively, while the typical dwell time for optical imaging is about 1000–6000 ns. Therefore, the differential traveling times of optical and acoustic will not impact on the scan rate of the PA system, and it allowed us to create the photoacoustic and optical image of NAD(P)H simultaneously at common scanning speeds (Fig. 6). To our knowledge this is the first NAD(P)H photoacoustic image and the first demonstration to penetrate deep into brain tissue and cerebral organoids without a significant loss of NAD(P)H detection signal, which is a key advantage of the photoacoustic approach. We believe this depth-resolved capability allows for unprecedented insights into the metabolic state of neurons and astrocytes within complex tissue architectures, facilitating studies of brain function in health and disease.

Although we succeeded in imaging endogenous NAD(P)H at single-neuron resolution (Fig. 6), it is still relatively low resolution compared to an all-optical microscope (Fig. S4b), as we chose a low NA objective lens to create a larger focal volume in order to keep the acoustic signal at or below 100 MHz in our LF-MP-PAM. High-frequency (hundreds of megahertz) acoustics suffer from significant signal loss due to low penetration. The development of a concave high NA acoustic lens might be an option to improve the spatial resolution^43^.

Another technical limitation of the current photoacoustic imaging system is that it is configured as a through-imaging system, which means the acoustic transducer is installed under the specimen (opposite side from the objectives). Another limitation of our system is that the transducer needs to be in physical contact with the sample through a coupling medium (water). A through-imaging system is undesirable or impractical for many clinical and pre-clinical applications^44^. To overcome these limitations, we would configure the laser and the photoacoustic transducer on the same side of the sample^3^ or mouse^45^, integrated with a photoacoustics sensor^46,47^ for in vivo imaging and human clinical application.

In our work, we did not distinguish NADH photoacoustic signals between neurons and astrocytes. In the future, we will endeavor to endeavor to differentiate the signals from multiple types of cells by using deep-learning-based classification^48,49^ along with computational enhancement of resolutions^50,51^. Our approach can be applied not only for brain imaging but also in vivo imaging of other organs and tissues, as well as in vitro imaging with multiple types of organoids, such as liver^52^, kidney^53^, and pancreas^54^ as well as ex vivo human embryo development^55^. In addition, our photoacoustic and optical hybrid-imaging can easily be combined with other natural metabolic biomarkers or genetically coded biosensors (e.g. GCaMP and jRGECO), ATP sensor (e.g,. GRAB-ATP^56^ and iATPSnFR^57^), Peredox sensor^58^, and voltage indicators (e.g., JEDI-2P^59^, ASAPs^60^, and Voltron^61^).

In summary, our photoacoustic imaging system (LF-MP-PAM) with a near-infrared laser, enables the non-invasive and label-free NAD(P)H detection relatively deep within brain slices and cerebral organoids while preserving spatiotemporal resolution. This approach provides a powerful tool for monitoring the metabolic dynamics of cells both in vitro and in vivo and label-free measurement of brain activity. This approach may be useful in both humans and animals to understand not only normal but also pathological mechanisms underlying neurodegenerative diseases and psychiatric disorders.

## Materials and Methods

### Development of photoacoustic imaging system with NIR laser

To demonstrate NAD(P)H detection by Photoacoustic signal and autofluorescence, photoacoustic sensors were fully integrated with custom-made three-photon microscope with a Spirit-NOPA laser system (30–40 fs, Spectra-Physics, see Fig. S1a). The 1300 nm wavelength laser was pumped up through white light supercontinuum generation and compressed from a 1045 nm Spirit-one laser (300 fs, 400 kHz, 16 W, Spirit, Spectra-Physics). The laser beams were scanned by a pair of galvo-galvo scanner (6215 H, Cambridge Technologies) to image and deliver the energy for photoacoustic generation on the back aperture of the objective using a pair of custom-designed scan (Thorlabs, SL50-3P, EFL: 50 mm, WD: 26.4 mm) and tube lenses (Thorlabs, TL200-3P, EFL: 200 mm, WD: 180.9 mm, ARC: 900 - 1900 nm). Automated image acquisition, control of scanners, and sample stage were carried out using ScanImage (ScanImage Premium 2023.0.0, Vidrio)^62^. A pair of collection lenses for four photon-multiplying tubes (PMT) collected the emitted signal from the sample. We typically ran either 256 × 256 or 128 × 128 pixels with 5000-10000 ns pixel dwell time and 0.524 (2 Hz) and 0.131 seconds per frame, respectively. The maximum field of view is 14.1 × 14.1 mm by the specs of the angle of the galvo-galvo scanner and scan lens, and the field of view was adjusted by zoom level from ScanImage.

The pulse power was adjusted by the pair of half-waveplates on the rotary mount (Fig. S1). The power of the polarized laser modulated at the first half-wave plate was reduced according to the angle of the second half-wave plate. The laser rep rate was adjusted from 1000 Hz to 200 kHz depending on the experiment. The pulse power was determined by measuring the average power at the objective with a thermopile power meter (Thorlabs, S370C with PM100D).

For optical imaging, all the fluorescent emission light was initially separated by a primary dichroic short-pass filter mirror (890 nm, Fig. S1a). NADH fluorescent signals were detected using GaAsP photomultiplier tubes (H7422A-40, Hamamatsu, Japan) with a band-pass filter (Semrock, 475/35 nm). The third harmonic Generation (THG) signal was detected using a bialkali (BA) photomultiplier tube (R7600U-200) and a narrow-bandwidth bandpass filter (Semrock, 400/10 nm). All the PMT signals were amplified (Hamamatsu C9999-01) and recorded through a National Instruments DAQ board (PXIe-6356 in PXIe-1073 chassis, 250 Ks/sec) and processed in ScanImage for image creation. Laser power can be attenuated by a polarizer down to 10 mW on surface observation and 35-45 mW for deep tissue. A Mitutoyo NIR objective lens (MY20X-824, 20x, N.A.: 0.4, W.D. 20 mm) was used for almost all photoacoustic experiments, and Olympus 20x objectives (Olympus XLPLN25XWMP2, NA: 1.05). Assuming the objective is a circular aperture, then at any axial position (z) the laser intensity is given by the Bessel function^63^. Automated image acquisition, control of scanners, and the sample stage were manipulated using ScanImage. The sample was placed on a x-y motorized stage, and the objective lens was placed on a single-axis motorized stage (MMBP, Scientifica) to move it in the x-y-z direction.

The photoacoustic sensor was placed just underneath the specimen (NAD(P)H in gel tissue phantom, cultured cells, brain slice, and cerebral organoid, Fig. S1a). We mostly used an Evident transducer (Center frequency: 125 MHz, bandwidth: 68-197 Hz, V2062, Olympus) to capture acoustic waves, but another Evident transducer (Center frequency: 75 MHz, bandwidth: 45-122 Hz, V2025, Olympus) was used only for the HepG2 uptake experiment. Both transducers have a 0.125-inch element in diameter. The cultured cells on the glass-bottom dish were placed directly on the transducer, and water was placed in between the bottom glass dish and the transducer element. Photoacoustic signals were then amplified by UT340 pulser receiver system (UTEX Scientific) using a high-pass filter of 20 MHz, a low-pass filter of 150 MHz, and a gain of 63 dB, then recorded by an oscilloscope (Tektronix, MDO oscilloscope at 500 Ms/s). In most instances, the signal was averaged over 250 laser pulse durations (approximately 10 μs). This has the advantage of reducing the noise significantly, but also has the disadvantage of averaging many dark pixels not containing neurons with those generating the signal, and thus reducing our signal. To detect the laser trigger for the oscilloscope synchronization of scanning, a photo diode was placed on another optical light pass from NOPA (Signal laser path, 860 nm, Fig. S1b).

To achieve real-time photoacoustic imaging, we installed a high-speed data acquisition board, vDAQ (2.5 Gs/s, MBF Bioscience). The photoacoustic signal after the amplifier was connected to the high-speed input channel of vDAQ, and the raw acoustic signal was recorded at a 2.0 GHz sampling rate (Fig. S6a). In order to synchronize the laser pulse and galvo-galvo scanning and to set time windows on signal condition, the photodiode signal was conditioned by a pulse stretcher to stretch the pulse width from 20 ns to 2.5 μsec (50% duty cycle at 200 kHz laser rep rate), the stretched TTL signal was input to clock a multiplier board to export the locked TTL signal at 8 MHz for external trigger for synchronization and 200 kHz for phase shift adjustment. Then, both TTL signals were introduced to trigger the line of vDAQ so that the sample clock of the acquisition DAQ can be synchronized to an external clock (e.g., the laser sync). Synchronizing to the laser clock will ensure a constant number of laser pulses/pixel in the scanning. process the photoacoustic and optical signals and create images (Fig. S6b). A time window was set at approximately 2.6 μs after laser trigger (Fig. S6b). Multiple photoacoustic pulses (4 laser pulses/pixel in each time window) were averaged in the window, and a grey value was calculated for the pixel value, and these were processed through all the pixels in real-time to create the image (Fig. S6c). Images in Fig. 6 and Fig. S6 were acquired with following parameters: Wavelength: 1300 nm, laser rep rate: 200 kHz, acquisition frequency 2.0 GHz, dwell time: 20 μsec, 256×256 pixels, 0.76 frame/sec, 4-8 frame average, laser power: 10–20 mW. Pixel resolutions are 0.9 μm/pixel and 2.1 μm/pixel for the cerebral organoid and the brain slice, respectively. Frame averaging was implemented to further reduce the noise. The photoacoustic image was denoised by deconvolution in MATLAB^64^, to improve the resolution to identify the single cell in the cerebral organoid (Fig. S6c).

### Signal processing of the acoustic signal

The acoustic signals acquired from the oscilloscope were further processed in MATLAB. To decompose the photoacoustic signals into distinct frequency bands, Continuous Wavelet Transform (CWT) and inverted CWT were performed within in package “Wavelet Toolbox”.

### Raw photoacoustic data recording for post processing

The photoacoustic signal from amplified were connected to a Pico Scope 6000 (Pico Technology) with 310 Ms/s sampling rate which provided raw acoustic signal, PMT, laser trigger and galvo positions respectively and recorded in a file on the computer for post processing. These data also were used in simulations to help determine the vDAQ window size and location for the optimal image generation (Fig. S6).

### Standard NADH in gel tissue phantom preparation

Standard NADH sample in gel was prepared using a cylindrical mold (3 mm in diameter, 1.58 mm in height) and with 10% gelatin from bovine skin, type B (Sigma, G9382) in distilled water combined with a stock solution of NADH (Millipore Sigma, N8129) to create the desired final concentrations.

### NADH incubation in HEK293T cells and HepG2

HEK293T (ATCC, CRL-3216) and HepG2 (ATCC, HB8065) cells were seeded onto a 35 mm glass bottom dish (Matek, with No. 1 glass, P35G-1.0-14-C) and cultured in DMEM (ThermoFisher Scientific, 10569010) supplemented with 10% heat inactivated Fetal Bovine Serum (ThermoFisher Scientific, A5670201) and 1% Penicillin and streptomycin. After 48 hours from cell seeding, NADH at 150 μM (final concentration) was added and incubated for 30 min. Subsequently, the cells were washed with DPBS (ThermoFisher Scientific, 14190144) three times and photoacoustic measurement was performed. All the cells were cultured in a incubator at 37 °C and 5% CO_2_ concentration.

### Brain slice preparation

To detect NADH photoacoustically in a brain slice, a mouse brain slice was prepared following procedure^65^. Briefly, wild-type C57BL/6 or CaMKII-Cre × GCaMP6s C57BL/6 mice are fully anesthetized with isoflurane and then euthanized by decapitation. The scalp and skull overlying most of the brain were removed, and then the whole brain was extracted from the skull and submerged in artificial cerebral spinal fluid (aCSF) solution supplemented with sucrose. The whole brain is further cut to remove the prefrontal cortex and cerebellum, and then the dissected brain is mounted on the stage of a vibratome. Brain slices were cut by the vibratome into slices 200 μm for standard thickness (Figs. 4, S5), or 1000 μm for a thick brain slice (Fig. 5)) in a 95% oxygen and glucose-rich environment. The entire anesthesia, decapitation, and brain removal takes less than 5 minutes to improve the viability of cells for the following experiment. We ensure that animals are deeply anesthetized before decapitation by noting the absence of response to vigorous paw pinch and involuntary auditory startle. After dissecting out the slice, they were kept in an oxygen and glucose-rich environment (in aCSF) at 4 °C for 30–60 min to recover from the shock and photoacoustic imaging was performed. For the glucose depletion experiment, the brain slice was placed in aCSF without glucose in an incubator at 37 °C and 5% CO_2_ concentration for 24 hours.

#### Cerebral organoid formation and differentiation

Human iPS cells were obtained by ALSTEM (iPS11, Episomal, HFF). The iPS cells were maintained on Matrigel-coated 6-well plates in mTeSR plus medium (STEMCELL Technologies, #100-0276) with 10 μM Y-23632 (Rock inhibitor, STEMCELL Technologies, #72302) for the first day after passages and split every 5 days using ReLeSR (STEMCELL Technologies, #100-0483). For 3d culture, the iPS cells were dissociated into single cells with TrypLE Express (ThermoFisher Scientific, 12604013) and then plated at 30,000 cells per each well of U-bottom ultra-low attachment 96-well plate (Corning, #7007) with mTeSR plus supplemented with 10 μM of Y-23632. After 24 h, the culture medium was replaced with neural induction medium knockout DMEM-F12 (ThermoFisher Scientific, 12660012), 15% (v/v) knockout serum replacement (ThermoFisher Scientific, 10828028), 1% (v/v) MEM-NEAA (ThermoFisher Scientific, 11140050), 1% (v/v) Glutamax (ThermoFisher Scientific, 35050061), 100 nM LDN-193189 (Millipore Sigma, SML0559), 10 μM SB431542 (Millipore Sigma, 616461), and 10 μM XAV939 (Millipore Sigma, X3004) and changed every other day. After 10 days of culture, the culture medium was replaced with 1:1 mixture of knockout DMEM/F12 and Neurobasal Plus medium (ThermoFisher Scientific, A3582901) supplemented with 0.5% (v/v) N2 supplement (ThermoFisher Scientific, 17502048), 1% (v/v) B27 plus supplement with vitamin A (ThermoFisher Scientific, A3582801), 1% (v/v) Glutamax, 1% (v/v) MEM-NEAA, 2.5 ng/ml (v/v) human insulin solution (Millipore Sigma, I9278), and the culture medium was changed every other day until 18 days. After 18 days of culture, culture medium was replaced with maintenance medium (Neurobasal medium supplemented with 1% (v/v) N2 supplement, 2% (v/v) B27 supplement without vitamin A, 1% (v/v) Glutamax, 1% (v/v) MEM-NEAA, 200 μM ascorbic acid, 100 M dibutyryl-cAMP (STEMCELL Technologies, # 73882), 2.5 ng/ml (v/v) human insulin solution, and 1% (v/v) Penicillin/Streptomycin). Cerebral organoids were collected at D21 and D90 for photoacoustic imaging experiments.

#### Fluorescent-based measurement of NADH

To have a second assessment, the cells were dissociated with TrypLE Express, from the cultured condition, brain slice, and cerebral organoid after photoacoustic measurement. Then, the cells were incubated with 0.1% of NADH fluorescent sensors (AAT Bioquest, JZL1707 NAD(P)H sensor)^29–32^ and were incubated for 30 min and washed with PBS for three time. Then, flow cytometry was performed with FACSMelody (BD) to quantify the level of NA(P)H in the cells.

#### Data processing of Ca^2+^ imaging data

Time-lapse images were first analyzed by Suite2p package on (suite2p, python = 3.9) to identify the signal neurons by determining regions of interest (ROIs). For each ROI time series, baseline fluorescence was defined as the average of the lowest 10% of samples. ∆F/F was computed as (F−F(average)/F_average_ and photo-bleaching was also normalized by the slope calculated by F_end_ -F_start_. From Exported data (delta F) the correlation matrix was calculated with Pearson’s pairwise correlation to estimate connectivity between neurons in MATLAB.

### Penetration depth model

The penetration depth model relied on the extinction length of 250 μm at 1300 nm, and photon absorption per pulse per molecule was reported^66^ for the 3-photon laser. A quantum yield of 5% was assumed in the simulations used for NAD(P)H, and an estimate of the 3-photon cross-section was made based upon a simple ratio of GCaMP (3-photon cross-section) x NAD(P)H (2-photon)/GCaMP (2photon) = 4.29 × 10^−85^. The photoacoustic transducer was characterized by a sensitivity of 140 μv/PA before the amplifier, whose gain was 1412.

#### Statistical Analysis

The reported values are the means of a minimum of three independent experiments. Data are presented as the mean ± Standard Deviation (SD). For equal variances and normality distribution, a Student’s t-test was performed. To compare groups at multiple conditions, statistical comparisons were performed using one-way analysis of variance (ANOVA), with post hoc pairwise comparisons carried out using the Tukey-Kramer method. Statistical tests were performed using GraphPad Prism 9 (GraphPad Software, San Diego, CA). *p* values < 0.05 or *p* values < 0.01 were considered significant in all cases.

## Supplementary information

Supplemental information

## Data Availability

Raw data reported in this paper is available from the corresponding author upon request. The MATLAB codes for image analysis and k-wave simulation are available from GitHub (https://github.com/surlab/lf-mp-photoacoustic-imaging).
