# Supplementary material for: Multi-photon, label-free photoacoustic and optical imaging of NADH in brain cells
Source: Light Sci Appl. 2025 Aug 7;14:264. doi: 10.1038/s41377-025-01895-x (PMC12331929; doi:10.1038/s41377-025-01895-x)
Supplement: Supplementary file 1 — Supplemental information [file 41377_2025_1895_MOESM1_ESM.pdf]

Supplemental information

Multi-photon, label-free photoacoustic and optical imaging of NADH in brain cells

Tatsuya Osaki[1, 2]†, W. David Lee[3]†, Xiang Zhang[3, 4]†, Rebecca E. Zubajlo[3]†, Mercedes Balcells[4], Elazer R. Edelman[4], Brian W. Anthony[3, 4]*, Mriganka Sur[1] *, Peter T. C. So[3, 5]*

Affiliations

1. Picower Institute for Learning and Memory, Massachusetts Institute of Technology, Cambridge, MA, 02139, United States
2. Whitehead Institute for Biomedical Research, Massachusetts Institute of Technology, Cambridge, MA, 02142, United States
3. Department of Mechanical Engineering, Massachusetts Institute of Technology, Cambridge, MA, 02139, United States
4. Institute of Medical Engineering and Science, Massachusetts Institute of Technology, Cambridge, MA, 02139, United States
5. Department of Biological Engineering, Massachusetts Institute of Technology, Cambridge, MA, 02139, United States

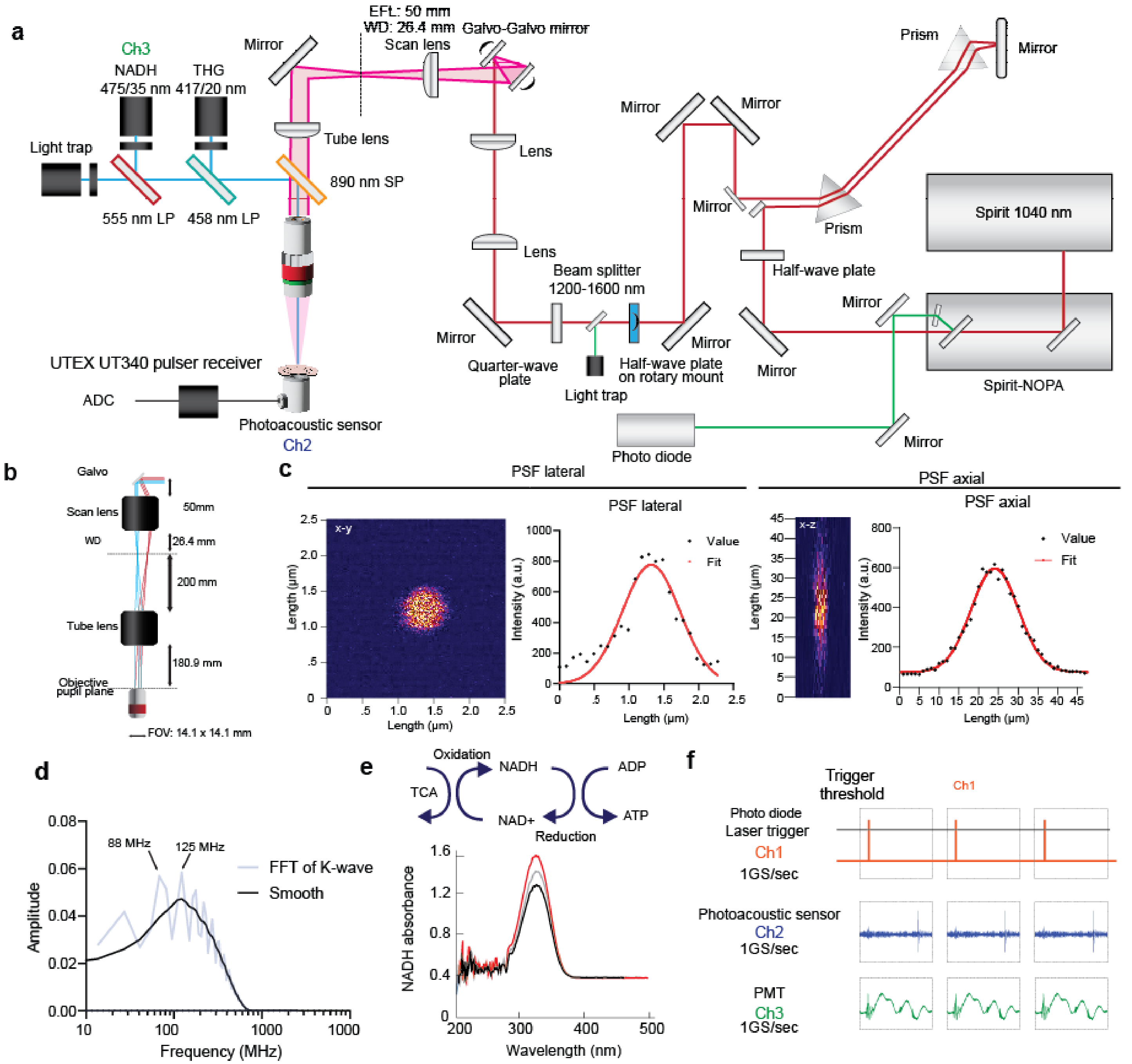

Figure S1 | (a) Details of the optical and photoacoustic setup. (**b**) Set up of scan lens and tube lens to get maximum resolution. (**c**) Measured point spread function (PSF) with 0.4NA. (**d**) K-wave simulation result for the heated volume with dominate peaks at 88 and 125 Mhz. (**e**) The metabolic cycle of creation and consumption of NAD(P)H and the measured 1-photon absorbance NAD(P)H. (**f**) Representative measuring of trigger signal, photoacoustic NAD(P)H signal, and optical NADH signal.

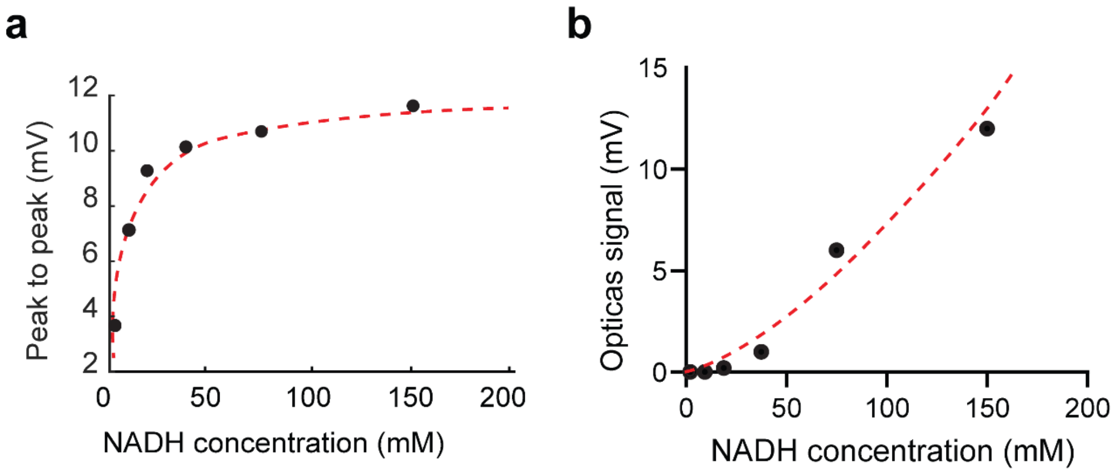

Figure S2| Photoacoustic signal vs concentration
(**a**) The photoacoustic NADH signal vs (**b**) optical NADH with multiple concentration (0- 150 mM). The linear concentration confirms that we are measuring NADH.

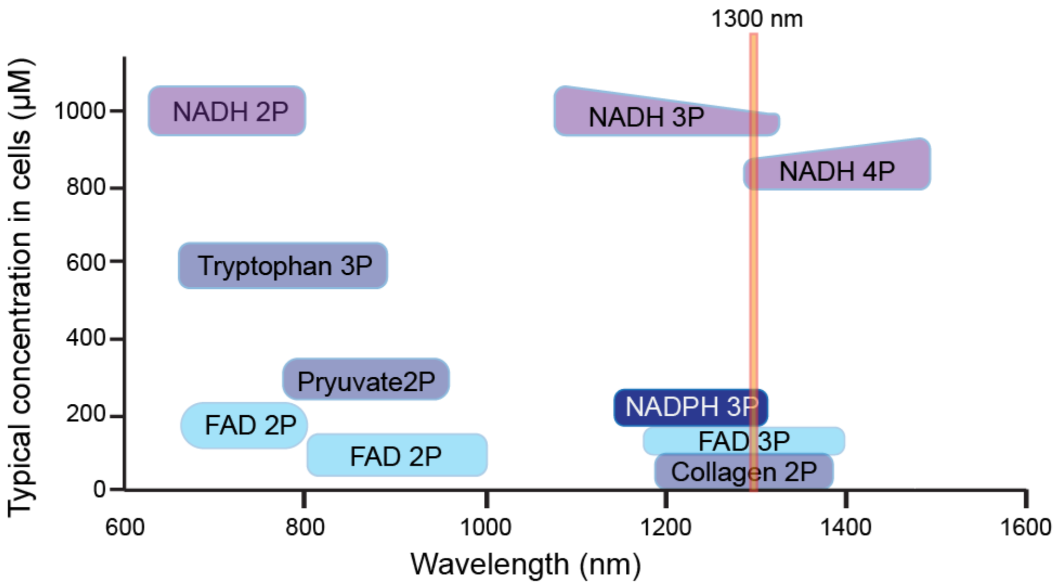

Figure S3| Optically active molecules of interest.
There are potential optical active molecules of interest at 600-1600 nm of excitation wavelength based on 1-photon and two-photon absorption. NADH is supposed to have strong peak at 700-720 nm at two-photon excitation, 1050-1200 nm at three-photon excitation, and 1300-1400 nm at four-photon excitation. Around 1300 nm wavelength, although NAD(P)H, FAD[1] (three-photon excitation) and collagen (two-photon excitation) potentially interfere with NAD(P)H (3P/4P), their intracellular concentrations are much lower than NAD(P)H in cells.

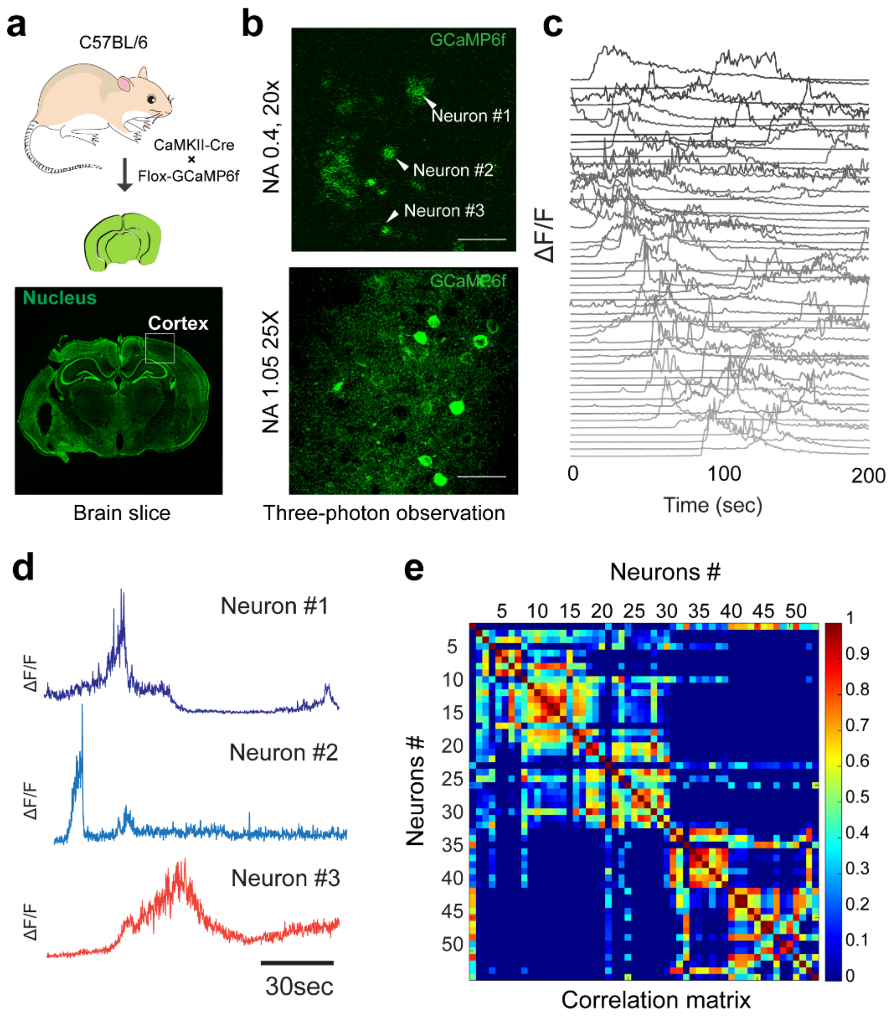

Figure S4| Verification of single neuron activity in mouse brain slice using GCaMP6 recording with 0.4 NA objective. (**a**) GCaMP6 mice brain slices were prepared and placed in the 3-photon microscope where the individual neuronal activity could be observed. (**b**) Comparison of resolution by two different NA of objectives. (**c**) Shows the signal from different neurons in a time series. The progression of the activity in the different neurons over 200 seconds suggests the interaction of the single neurons in the brain slice. (**d**) Calcium traces showing activity of representative single neurons. (**e**) Correlation matrix indicates the high degree of interaction of the individual neurons.

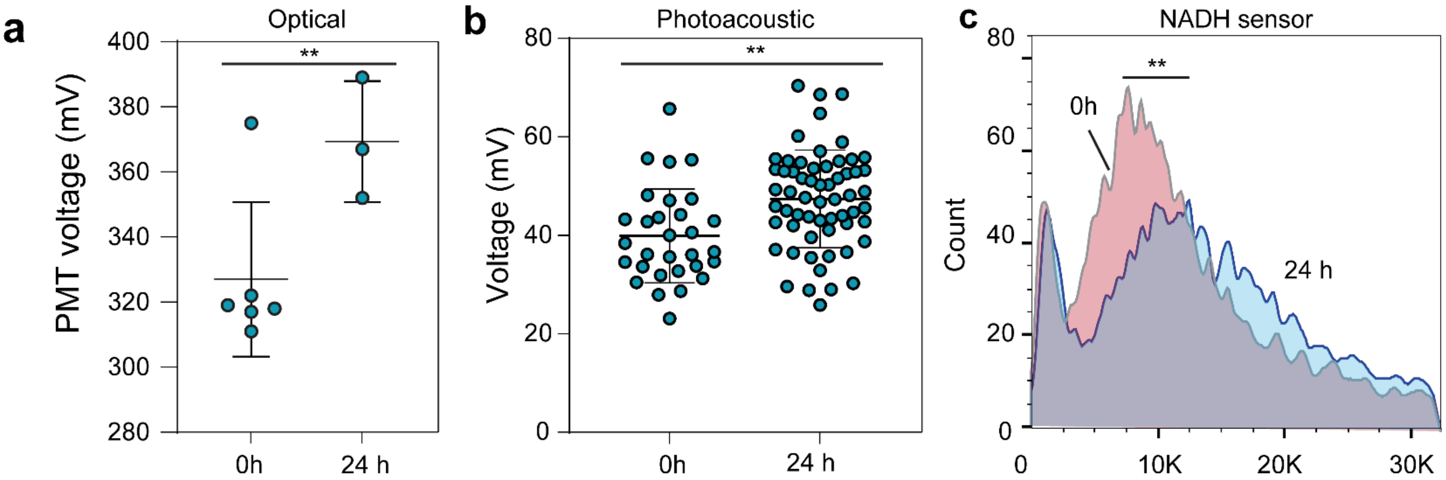

Figure S5| Optical, photoacoustic, and flow cytometry measurements of NAD(P)H during metabolic changes from 24-hour reduced glucose. (**a**) NAD(P)H variation by reduced glucose and oxygen for 24 hours. n = 3 different samples (**b**) Photoacoutic recording of NAD(P)H immediately after preparation with 95% oxygen at time 0 and after 24 hours in the air (hypoxia condition) and glucose withholding. n = 10. (**c**). Flowcytometry (count vs fluorescent signal) with commercially available NAD(P)H sensor of brain slices on day 0 and after 24 hours. The area under the curve of hypoxic neurons shows a 46% increase of NADH confirming the increase in NAD(P)H measured by the photoacoustic signal. **, $p<0.01$, Student's t-test

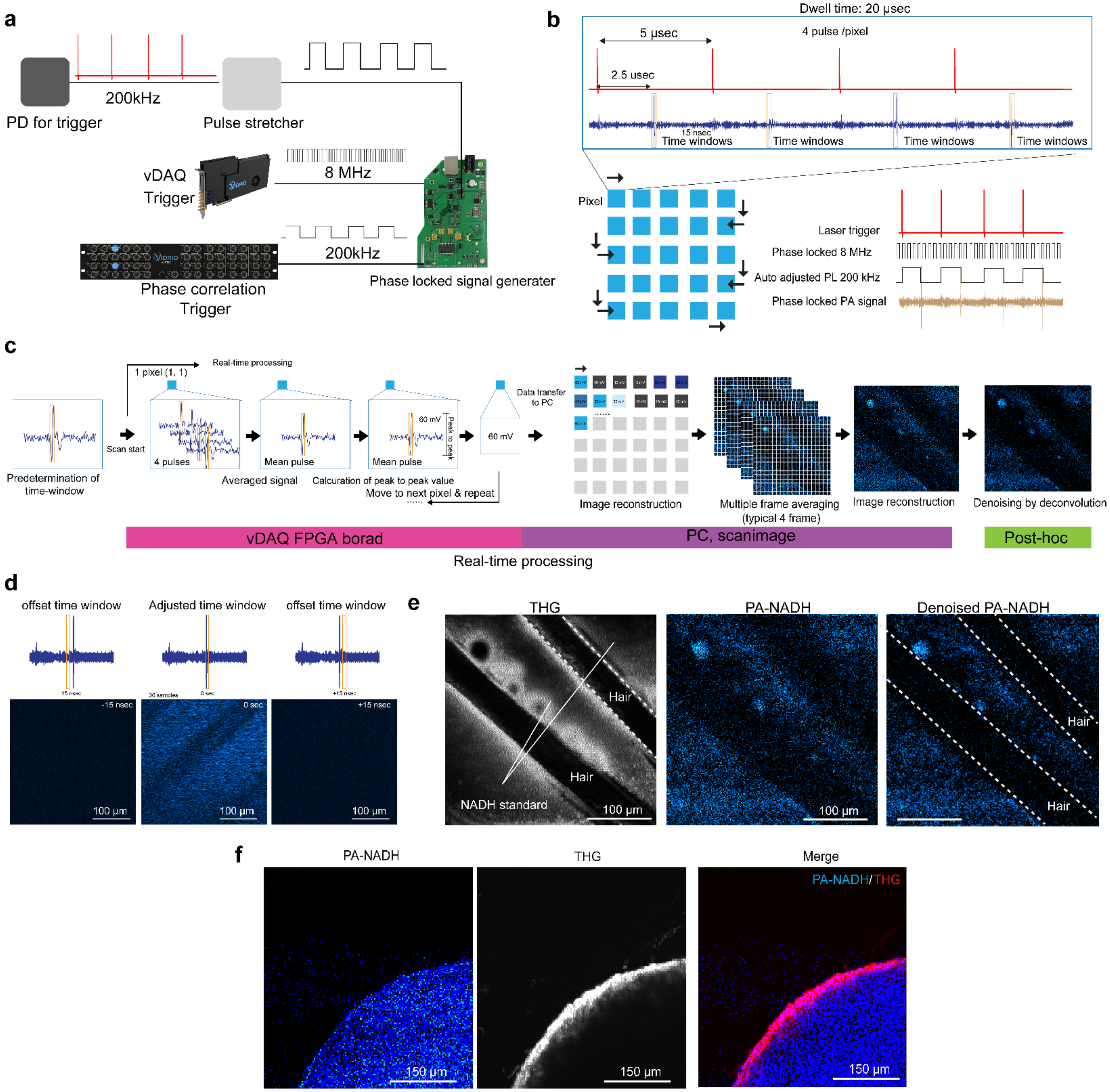

Figure S6| Photoacoustic imaging setup.

(**a**) To achieve synchronization of scanning and laser pulse and perform photoacoustic imaging, the photodiode (located on the branched light-path) electrical signal was stretched at 50% duty cycle with a custom pulse stretcher, then signal was input to phase locked signal generator to have 8 MHz laser-phase locked signal for vDAQ trigger and 200 kHz for phase correlation trigger. (**b**) For photoacoustic image creation, the galvo-scanner was run at 20 µsec dwell time, which means 4 laser pulses were emitted during parking one pixel. Because photoacoustic wave should be captured after 2.6 µsec from laser trigger, the acquisition window (15 nsec) was set after 2. 6 µsec during real-time scanning. (**c**) Flow of signal processing from photoacoustic signal detection to recreation of photoacoustic image. (**d**) Images were obtained when the acquisition window was set to capture the photoacoustic wave, otherwise images were lost. (**e**) Representative image of photoacoustic image for NAD(P)H standard with hair, along with all-optical imaging (THG). We believe the bubbles are heating of the melanin in the hair (i.e. at the interface) which generates a cavity of gel/water near the hair. (**f**) NAD(P)H photoacoustic

imaging of 3 month-cultured cerebral organoids (PA-NAD(P)H) along with THG and optical NADH. Photoacoustic imaging of cerebral organoids with PA-NADH and THG showed overlapped signals and matched field of view. Wavelength: 1300 nm, laser rep rate: 200 kHz, acquisition frequency 2.0 GHz, dwell time: 20 μsec, 256×256 pixels, 0.76 frame/sec, laser power: 10-20 mW. Pixel resolution is 0.9 um/pixel. (d, e)

1 Huang, S., Heikal, A. A. & Webb, W. W. Two-photon fluorescence spectroscopy and microscopy of NAD(P)H and flavoprotein. *Biophys J* **82**, 2811-2825 (2002). https://doi.org:10.1016/S0006-3495(02)75621-X
